# Mechanisms underlying dental-derived stem cell-mediated neurorestoration in neurodegenerative disorders

**DOI:** 10.1186/s13287-018-1005-z

**Published:** 2018-09-26

**Authors:** Syed Shadab Raza, Aurel Popa Wagner, Yawer S. Hussain, Mohsin Ali Khan

**Affiliations:** 1 Laboratory for Stem Cell & Restorative Neurology, Department of Biotechnology, Era Medical College & Hospital, Era University, Lucknow, Uttar Pradesh 226003 India; 2 Departmentof Dental Materials, RUHS College of Dental Sciences, Subhash Nagar, Jaipur, Rajasthan 302002 India; 3 Department of Neurology, Chair of Vascular Neurology and Dementia, Essen University Hospital, Essen, Germany; 4 Era Medical College & Hospital, Era University, Lucknow, Uttar Pradesh 226003 India; 5 Department of Stem Cell Biology and Regenerative Medicine, Era University, Lucknow, 226003 India; 6 Center of Clinical and Experimental Medicine, University of Medicine and Pharmacy Craiova, Craiova, Romania; 7 School of Medicine, Griffith University, Southport, QLD Australia

**Keywords:** Dental-derived stem cells, Cell replacement, Paracrine effect, Vasculogenesis, Synaptogenesis, Immunomodulation, Apoptosis

## Abstract

**Background:**

Neurodegenerative disorders have a complex pathology and are characterized by a progressive loss of neuronal architecture in the brain or spinal cord. Neuroprotective agents have demonstrated promising results at the preclinical stage, but this has not been confirmed at the clinical stage. Thus far, no neuroprotective drug that can prevent neuronal degeneration in patients with neurodegenerative disorders is available.

**Main body:**

Recent studies have focused on neurorestorative measures, such as cell-based therapy, rather than neuroprotective treatment. The utility of cell-based approaches for the treatment of neurodegenerative disorders has been explored extensively, and the results have been somewhat promising with regard to reversing the outcome. Because of their neural crest origin, ease of harvest, accessibility, ethical suitability, and potential to differentiate into the neurogenic lineage, dental-derived stem cells (DSCs) have become an attractive source for cell-based neurorestoration therapies. In the present review, we summarize the possible use of DSC-based neurorestoration therapy as an alternative treatment for neurodegenerative disorders, with a particular emphasis on the mechanism underlying recovery in neurodegenerative disorders.

**Conclusion:**

Transplantation research in neurodegenerative diseases should aim to understand the mechanism providing benefits both at the molecular and functional level. Due to their ease of accessibility, plasticity, and ethical suitability, DSCs hold promise to overcome the existing challenges in the field of neurodegeneration through multiple mechanisms, such as cell replacement, bystander effect, vasculogenesis, synaptogenesis, immunomodulation, and by inhibiting apoptosis.

## Background

Neurodegenerative disorders caused by neurodegeneration encompass a broad range of diseases of the central nervous system (CNS) and peripheral nervous system (PNS) and affect tens of millions of people worldwide [1]. Neurodegeneration is a progressive and irreversible loss of neuronal structure and function; it can be acute (e.g., stroke and spinal cord injury (SCI)) or chronic (e.g., Alzheimer’s disease and Parkinson’s disease).

Currently, considerable neurological research is focused on methods for regenerating and replacing the degenerated nerve cells; thus, stem cell therapy may be the most suitable clinical intervention for neurodegenerative disorders. The nervous system has limited intrinsic repair ability, because the endogenous population of neural stem (or progenitor) cells is so small that it can barely contribute to the structural repair of the brain or spinal cord [2–5]. Thus, therapies using exogenous stem cell sources may aid in alleviating various neurological diseases [6]. However, the most suitable cell type and the accurate timing and route of delivery need to be defined; most importantly, how a functional improvement from the behavioral perspective can be achieved remains unanswered [7, 8].

**Fig. 1:**
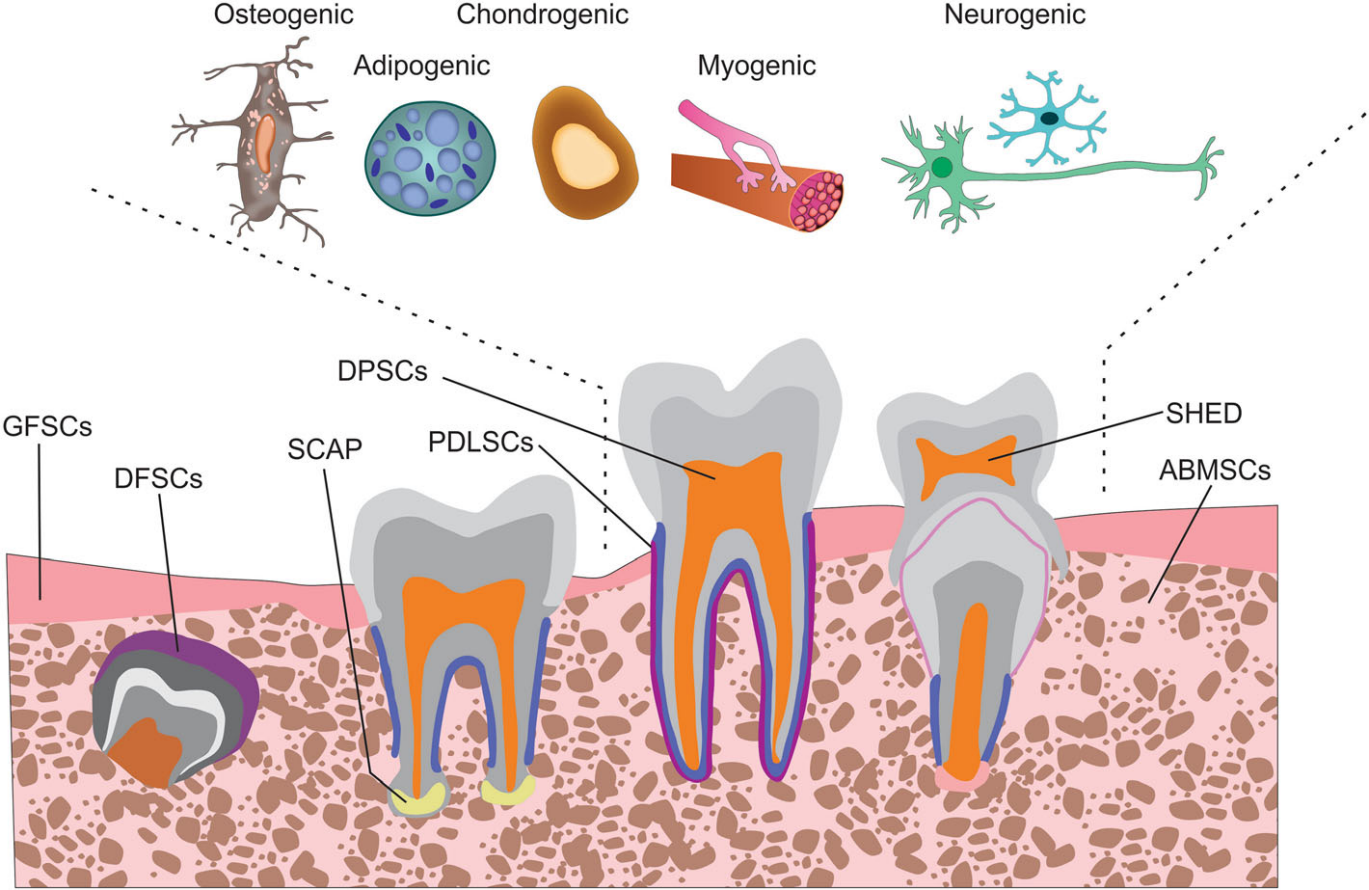
The transformation of dental tissue into different dental-derived stem cell populations. Subpopulations can be categorized according to their tissue of origin: dental pulp stem cells (DPSCs), stem cells from human exfoliated deciduous teeth (SHEDs), stem cells from the apical papilla (SCAPs), tooth germ progenitor cells, gingival mesenchymal stem cells, dental follicle stem cells (DFSCs), alveolar bone-derived mesenchymal stem cells (ABMSCs), gingival fibroblastic stem cells (GFSCs), and periodontal ligament stem cells (PDLSCs). The differentiation potential of dental pulp stem cells into various cell types illustrating the plasticity of bone marrow-derived cells is illustrated above

**Fig. 2:**
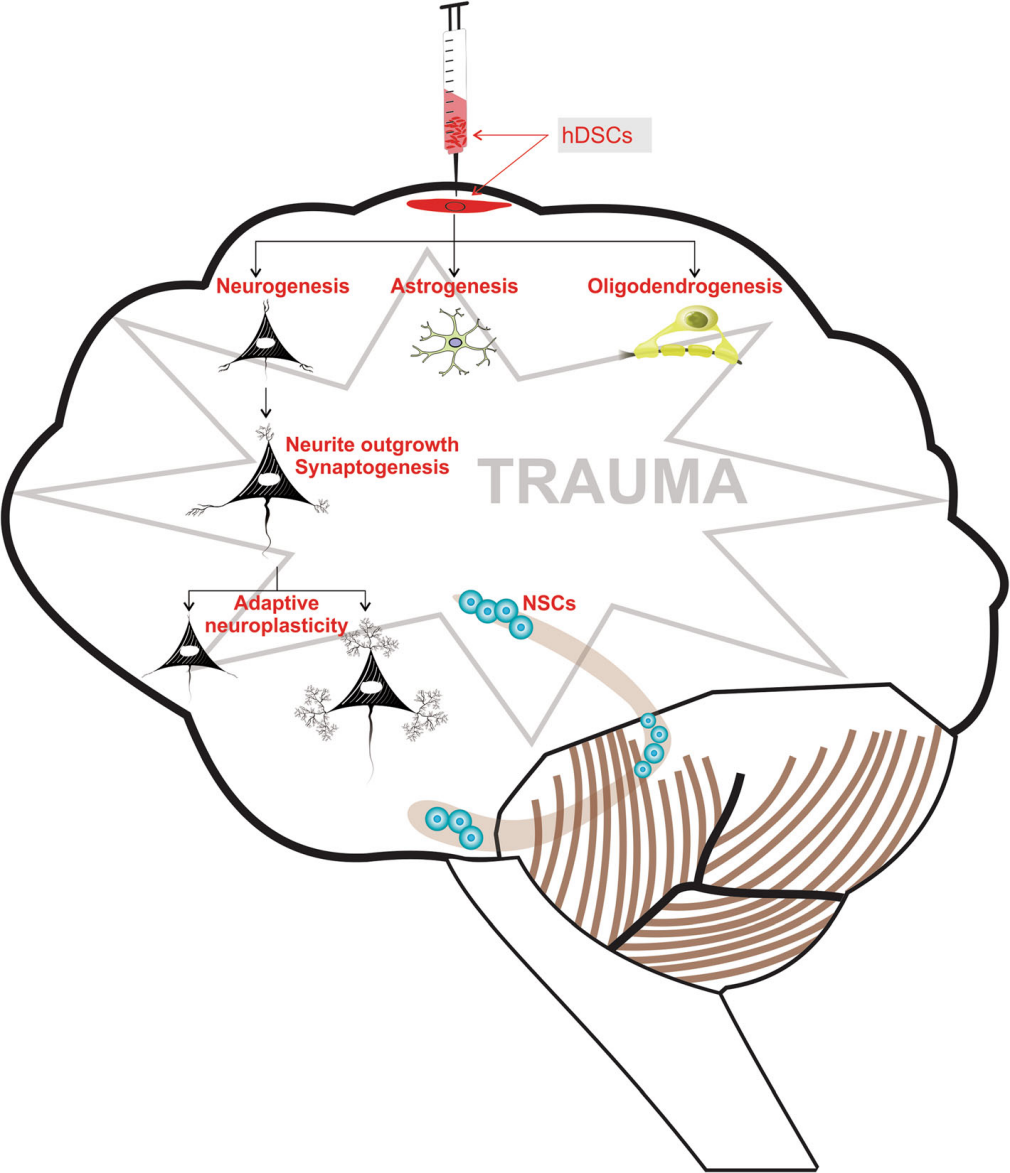
The transdifferentiation-mediated neuroplasticity mechanism of dental derived stem cells in neurological diseases. hDSC human dental-derived stem cell

For cell therapy, various types of stem cells (e.g., embryonic, fetal, adult, and induced pluripotent cells) can be obtained from various sources (e.g., the heart, skin, liver, and hair). Regardless of their type and source, all stem cells possess indefinite self-renewal capacity and can differentiate into a specialized cell type [9]. In 2002, dental-derived stem cells (DSCs) were first isolated from the pulp of permanent teeth by Gronthos et al. [10] and were named dental pulp stem cells (DPSCs), indicating that dental tissue can be a potential source of stem cells. Later, stem cells from human exfoliated deciduous teeth (SHEDs) [11], stem cells from the apical papilla [12], tooth germ progenitor cells [13], gingival mesenchymal stem cells (MSCs) [14], dental follicle stem cells [15], alveolar bone-derived MSCs (ABMSCs) [16], and periodontal ligament stem cells (PDLSCs) [17] were isolated and characterized (Fig. 1). Because of their transdifferentiation ability, DSCs have been extensively investigated and have shown high potential for application in CNS therapy [18–22]. Cell-based and preclinical studies have demonstrated that DSCs display neuroplasticity; they can differentiate in response to environmental cues into various cell lineages, such as adipogenic [23], chondrogenic [10], osteogenic [24], myogenic [25], and neurogenic lineages [26] (Fig. 1). DSCs have shown a migratory capacity toward the sites of neural damage where they differentiate into neurons [27], glia [28], and oligodendrocytes [20] as per the environmental cues, and they stimulate endogenous neurogenesis [29] and restore synaptic transmission [30]. Transplanted DSCs exhibit the restoration of functional outcome in rodents [20, 21]. Together the cellular and molecular data indicate that DSCs have the capacity to restore neuroplasticity after differentiation (Fig. 2). With regard to DSC application in dental therapy, at least one clinical trial has been completed and a few are ongoing (Table 1); however, no clinical trial on the neurological application of DSCs has been initiated thus far.

**Table 1:** Summary of the clinical effect of dental-derived stem cells (DSCs)

Clinical trial number	Study type	Phase	*n*	Disease type	Length of trial (months)	DSC type	Status	Observed changes	Reference
NCT03386877	Periodontal regeneration using DPSCs	_	29	Periodontal diseases	15 (January 2016 to April 2017)	DPSCs	Completed	Not known	[136]
NCT02523651	Periodontal regeneration of chronic periodontal disease patients receiving stem cell injection therapy	I and II	40	Periodontal diseases	24 (December 2014 to December 2016)	1×10^6^ DPSCs immediately after periodontal scaling and root planing	Unknown	Change from baseline alveolar bone volume	–
NCT01814436	Revitalization of immature permanent teeth with necrotic pulp using SHEDs	I	80	Permanent incisor avulsed by trauma	58 (February 2013 to October 2017)	SHEDs	Active	Pulp and apical regenerated	–
NCT02464202	Use of CBCT-based tooth replica in tooth autotransplantation to improve the outcome of tooth replacement in children	_	100	Increase success rate of tooth transplantation	56 (February 2013 to October 2017)	PDLSCs	Active	Not known	–
NCT02731586	Effect on allogenic MSCs on osseointegration of dental implants	Early phase I	10	Edentulous alveolar ridge	27 (January 2016 to March 2018)	Dental pulp-derived allogenic MSCs	Active	Not known	–
NCT02449005	Autologous ABMSCs for the reconstruction of infrabony periodontal defects (PerioRegen)	I and II	30	Chronic periodontitis	45 (January 2014 to September 2017)	ABMSCs	Active	Gain in clinical attachment level	–
NCT03137979	GMSC treatment of chronic periodontitis	I and II	30	Periodontitis	36 (January 2017 to December 2019)	GMSCs, collagen scaffolds, and open flap debridement	Active	An increase in the height of alveolar bone in mm	–
NCT01357785	Periodontal tissue regeneration using autologous PDLSCs	I	35	Periodontal pocket	32 (April 2011 to December 2014)	Autologous PDLSCs	Unknown	Increase in alveolar bone height and gain in clinical attachment level	[137]
NCT01082822	PDLSC implantation in the treatment of periodontitis	I and II	80	Chronic periodontitis	24 (January 2010 to January 201)2	PDLSC implantation (fabricated cell sheet pellets and cell sheet fragment)	Unknown	Not known	–

*ABMSC* alveolar bone-derived mesenchymal stem cell, *CBCT* cone beam computed tomography, *DPSC* dental pulp stem cell, *GMSC* gingiva mesenchymal stem cell, *MSC* mesenchymal stem cell, *PDLSC* periodontal ligament stem cell, *SHED* stem cell from human exfoliated deciduous teeth; *n* = no of participants

**Fig. 3:**
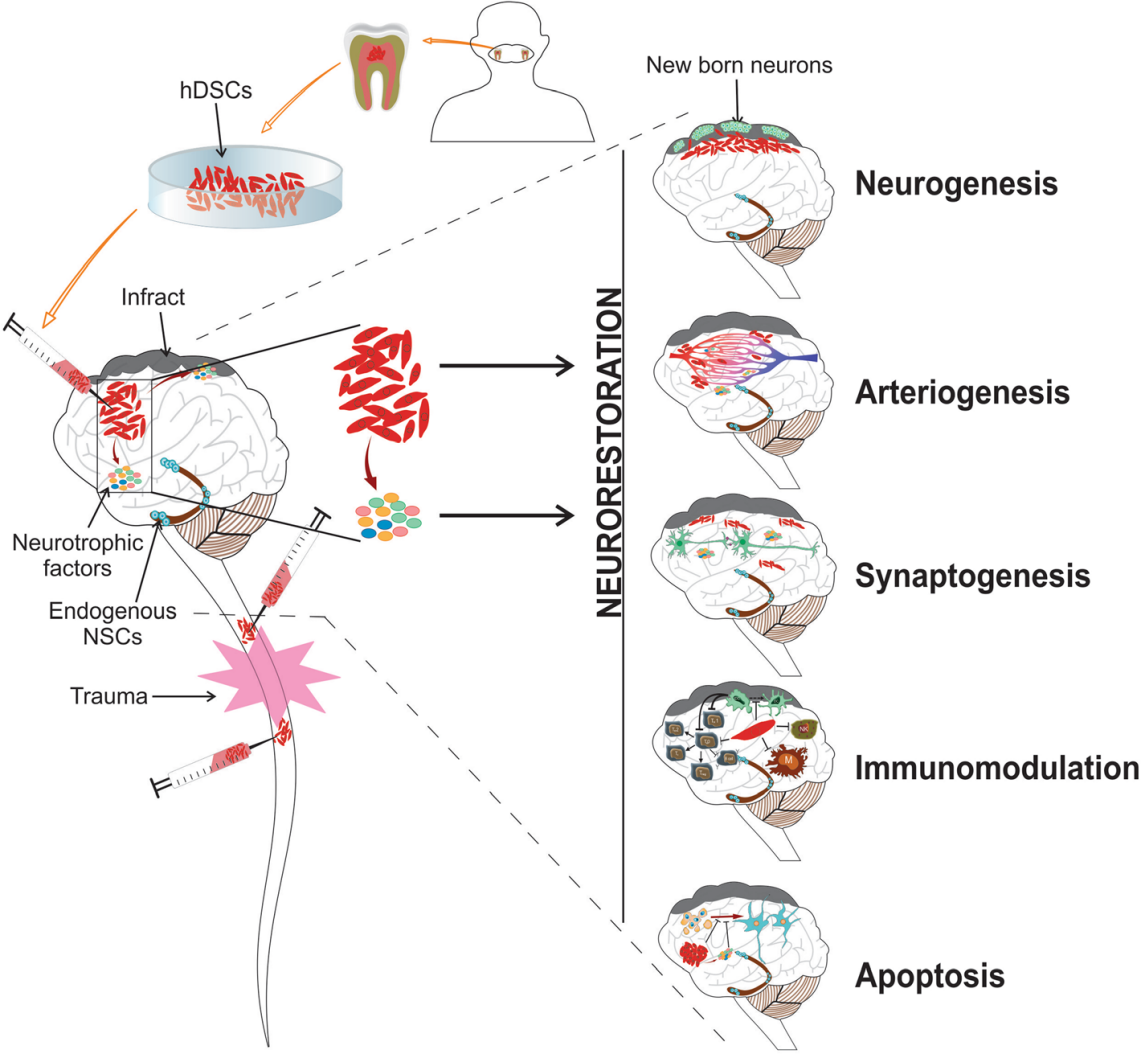
The mechanistic processes involved in dental-derived stem cell-induced neurorestoration in neurodegenerative disorders. Transplanted human dental-derived stem cells (hDSCs) activate an array of restorative events possibly through cell replacement, parenchymal secretion of growth and trophic factors, angiogenesis, immunomodulation, and by inhibiting apoptosis. The remodeling can be achieved most likely through bystander effects, except for the direct integration of the cells

The mechanism by which DSC transplants evoke CNS remodeling remains unknown. Nevertheless, the transplanted DSCs are assumed to differentiate and integrate into the damaged CNS [8] to provide protection at the cellular and molecular levels. However, recent evidence strongly suggests that a range of other neurorestorative factors, such as angiogenesis [31], synaptogenesis [32], immunomodulation [33], and apoptosis inhibition [34] (Fig. 3), along with neural replacement, contributes toward recovery.

In the present review, we focus on the therapeutic efficacy of the exogenous DSCs transplanted for treating neurodegenerative disorders in various models (Table 2). We also emphasize the probable mechanisms by which DSCs facilitate endogenous repair and plasticity in the CNS. Considering DPSCs and SHEDs, the two subtypes extensively studied and employed to study the neurological restorative measures of cell integration, angiogenesis, synaptogenesis, immunomodulation, and the apoptosis inhibition mechanism, we argue the advantages of using DSCs to treat various neurodegenerative disorders.

**Table 2:** Summary of dental-derived stem cell (DSC)-mediated neuroprotection

Neurodegenerative disease	Model type	Cell type	Mechanism of action	Model	Reference
Alzheimer’s Diseases	In vitro	DPSC	Promoted regeneration of neuron cells by inducing cell proliferation, reducing apoptotic cell death, prolongation of dendrites, and by inhibiting phosphorylation of tau protein	Okadaic acid induced Alzheimer’s disease in SH-SY5Y cells	[41]
			DPSCs cocultured with primary hippocampal and ventral mesencephalic showed high protection against β-amyloid protein by secreting neurological factors such as NGF, GDNF, BDNF, and BMP2	β-amyloid peptide (1–42)-treated primary culture of hippocampal neuron and mesencephalic cells	[42]
	In vivo	SHED	Serum-free conditioned medium derived from SHEDs improved overall cognitive function by axonal elongation, neurotransmission, suppression of inflammation, and by induction of anti-inflammatory M2-like microglia	Aβ1–40 peptide infused in imprinting control region (ICR) mice	[39]
Parkinson’s disease	In vitro	SHED	SHED-derived exosomes prevented apoptosis by suppressing caspase activity by approximately 80%	6-OHDA-induced apoptosis in ReNcell VM human neural stem cell-derived dopaminergic neurons	[34]
			Conditioned medium from SHED and, SHED derived dopaminergic neuron protected primary neurons against 6-OHDA toxicity and accelerated neurite outgrowth by paracrine mechanisms	Dopaminergic neuron	[45]
		DPSC	DPSC protected mouse dopaminergic neurons by the release of neurotrophins such as BDNF and NGF	MPP^+^- or rotenone-treated mesencephalic cells	[43]
			Human dental pulp cells attenuated 6-OHDA toxicity through expressing neuronal phenotype and releasing NGF, GDNF, BDNF, and BMP2	6-OHDA-treated primary culture of hippocampal neuron and mesencephalic cells	[44]
			DPSCs through their immunomodulatory properties attenuated the proliferation and production of ROS and NO	1-methyl-4-phenyl-1,2,3,6-tetrahydropyridine (MPTP)-treated coculture system of neuron and microglia	[28, 138]
	In vivo	SHED	Dopaminergic neurons derived from SHED expressed BDNF, GDNF, NT3, and HGF when transplanted in Parkinsonian rats and improved the dopamine level	6-OHDA-induced Parkinsonian rat	[45]
			SHED treatment prevented 6-OHDA-induced neuronal damage in rats contributing to the improvement of behavioral outcome. Cells showed neuronal and glial expression; moreover, SHED-derived differentiated spheres had a better outcome suggesting predifferentiation could be a key step for Parkinson’s Disease transplantation therapy	6-OHDA-induced Parkinsonian rat	[44]
Spinal cord injury (SCI)	In vitro	DPSC	DPSC-laden microcapsules transplanted into an organotypic SCI model; the cells survived for 10 days and demonstrated commitment to a neural lineage	Organotypic SCI model	[139]
	In vivo	SHED	SHED transplantation in traumatic SCI rats reduced the cystic cavity area and glial scar and increased the neurofilament along with lower expression of TNF-α	Traumatic SCI in Wistar rats	[47]
			SHED transplantation in SCI reduced early neuronal apoptosis, which contributed to tissue and motor neuron preservation and hindlimb functional recovery	Laminectomy followed by SCI in Wistar rats	[116]
			Conditioned serum-free medium from SHEDs into rat injured spinal cord during the acute postinjury period caused remarkable functional recovery which was attributed to the immunoregulatory activity that induced anti-inflammatory M2-like macrophages	Laminectomy followed by SCI in Sprague-Dawley rats	[117]
			SHEDs promote functional recovery when either SHED or SHED-induced neural cells were transplanted. The transplanted cells expressed neuronal and glial differentiation along with an increase in myelin basic protein and chondroitin sulfate proteoglycan NG2 and lower expression of GFAP	Laminectomy followed by SCI in Wistar rats	[48]
		DPSC	DPSC engraftment enhanced the number of surviving motor neurons in a hemisected spinal cord through secreting various neurotrophic factors, e.g., NGF, BDNF, and GDNF	Laminectomy followed by SCI in Sprague-Dawley rats	[84]
			DPSC inhibited the SCI-induced apoptosis of neurons, astrocytes, and oligodendrocytes, which improved the preservation of neuronal filaments and myelin sheaths. Paracrine mechanisms along with cell integration were the factors found in achieving recovery	Laminectomy followed by SCI in Sprague-Dawley rats	[20]
			DPSCs transplanted together with chitosan scaffolds resulted in the marked recovery of hindlimb locomotor functions. The levels of BDNF, GDNF, basic NGF, and NT3 were found to be significantly higher in the DPSC/chitosan-scaffold group	Laminectomy followed by SCI in Sprague-Dawley rats	[37]
			Significant improvement of limb function was observed when DPSCs were transplanted in dogs with chronic spinal cord injuries	Hemilaminectomy in dogs	[140]
			DPSCs demonstrated potential in repairing the completely transected spinal cord and promoting functional recovery after injury by inhibiting the expression of IL-1β, the expression of RhoA to promote neurite regeneration, and SUR1 expression to reduce progressive hemorrhagic necrosis, and by differentiating into mature neurons and oligodendrocytes	Laminectomy followed by SCI in Sprague-Dawley rats	[141]
Stroke	In vitro	DPSC	Human DPSCs showed superior neuroprotective, migratory, and in-vitro angiogenic effects versus human BMMSCs in a comparative study between the two cell types by blocking reactive gliosis, ROS production, and inflammatory mediators, e.g., IL-1 β	Oxygen–glucose deprivation (OGD)-injured human astrocytes	[36, 65]
	In vivo	SHED	Transplantation of SHEDs or the conditioned medium significantly improved the neurological outcome by inhibiting the expression of proinflammatory cytokines, e.g., TNF- α and IL-1 β, and apoptosis, and by enhancing the expression of anti-inflammatory cytokines, e.g,. IL-4, IL-6, IL-10, IL-13, and by reducing tissue loss	Hypoxia–ischemia brain injury was induced in postnatal day-5 mice	[55]
			SHED-derived conditioned medium enhanced neurogenesis, migration and differentiation of endogenous NPCs, induced vasculogenesis, and ameliorated ischemic brain injury after permanent MCAO	Permanent MCAO in Sprague-Dawley rats	[19]
		DPSC	Transplanted human DPSCs compared with human BM-MSCs in a rat stroke model had greater reduction in infarct volume. Administration of DPSCs to rats with stroke significantly decreased reactive gliosis compared with BM-MSCs	MCAO in Sprague-Dawley rats	[36]
			Dental pulp-derived side population stem/progenitor cells enhance recovery of transient focal cerebral ischemia in rats by promoting migration and differentiation of the endogenous neuronal progenitor cells and induced vasculogenesis	Transient MCAO in Sprague-Dawley rats	[53]
			Intracerebral transplantation of human DPSCs following focal cerebral ischemia in rats resulted in significant improvement in forelimb sensorimotor function at 4 weeks post-treatment through cell replacement and the paracrine effect	Transient MCAO in Sprague-Dawley rats	[21]

*6-OHDA* 6-hydroxydopamine, *BDNF* brain-derived neurotrophic factor, *BMMSC* bone marrow-derived mesenchymal stem cell, *BMP2* bone morphogenetic protein 2, *DPSC* dental pulp stem cell, *GDNF* glial cell-derived neurotrophic factor, *GFAP* glial fibrillary acidic protein, *HGF* hepatocyte growth factor, *IL* interleukin, *MCAO* middle cerebral artery occlusion, *MPP* 1-methyl-4-phenylpyridinium, *NG2* neural/glial antigen 2, *NGF* nerve growth factor, *NO* nitric oxide, *NPC* neural progenitor cell, *NT3* neurotrophin-3, *RhoA* Ras homolog gene family member A, *ROS* reactive oxygen species, *SHED* stem cell from human exfoliated deciduous teeth, *SUR1* sulfonylurea receptor 1, *TNF* tumor necrosis factor

## DSCs as a therapeutic choice in neurodegenerative disorders

Neurodegenerative disorders are heterogeneous and involve inter-related pathophysiological metabolic cascades, unlike an ideal clinical condition. However, for functional recovery, stem cell therapy for neurodegenerative disorders requires a cellular approach that has the potential to induce all neurorestorative processes. Various stem cell types are available for neurodegenerative therapy, including DSCs. The advantages of DSCs include that they are postnatal stem cell populations with MSC-like characteristics, including the capacity for self-renewal and multilineage differentiation, and this makes them a promising cell therapy candidate in neurodegenerative disorders; noninvasive isolation, ease of harvest, easy accessibility, and strong therapeutic ability are the key advantages of DSCs. They have no associated ethical concerns, which is a drawback often associated with other cell types such as induced pluripotent stem cells [35], though, they have high immunosuppressive activity [36, 37]. In the presence of specific stimuli, both DPSCs and SHEDs can differentiate into several brain cell types, including neurons and glia, thus indicating their neurogenic potential. Both DPSCs and SHEDs are derived from the neural crest, and thus have an origin different from bone marrow-derived MSCs (BMMSCs), which are derived from the mesoderm [38, 39]. Notably, DPSCs have clonogenicity and higher ex-vivo proliferative capacity [40, 41] compared with MSCs; they are less prone to malignancy [42], and thus can generate sufficient numbers of cells for cell therapy. DSCs have exhibited increased neurogenesis [40, 43], and these cells can influence endogenous stem cell recruitment and neurosphere generation [44, 45]. SHEDs are more developed and metabolically active than BMMSCs [46]. Compared with umbilical cord stem cells, DPSCs demonstrated delayed cellular senescence [47] which can be correlated to the increased expression of genes related to growth factors [48]. The beneficial effects of DPSCs and SHEDs on angiogenesis, neurotrophic secretion, and immunomodulation are well defined. Notably, these cells demonstrated targeted migration toward the lesion site [21, 49] which is also the therapeutic target. Furthermore, with improved dental hygiene, autologous transplantation of these cells is easy.

## DSCs and neurodegenerative disorders

The following sections discuss the potential use of DSCs in the treatment of neurodegenerative disorders. Brain and spinal cord disorders are characterized by neurodegeneration (the potential loss of neuronal architect and function) which cannot be adequately repaired by the host. In this context, DSCs have become a focus as a novel alternative to salvaging or reconstituting the lost architecture or to stimulating host repair [21, 50–52]. Because of their neural crest origin, the potential ability of DSCs to directly perform neuronal replacement has been recently explored; these cells could differentiate and integrate into the cells of the neuronal lineage in the CNS [22, 53]. However, the ability of DSCs to provide benefits by differentiating and integrating into the system has recently been challenged because, although DSCs integrate into the diseased brain or spinal cord, the number of transplanted DSCs is much lower than that required for the affected area. Thus, several other mechanisms, apart from cell integration, must be involved in the process of neurorestoration provided by these cells.

### Alzheimer’s disease

Alzheimer’s disease is an incurable, progressive, multifarious neurodegenerative disease. Thus far, no effective treatment to prevent, arrest, or reverse this disease has been reported. However, advances in understanding the etiology of the disease and routine research on new therapeutic measures have provided hope for improved Alzheimer’s disease management. Recent findings indicate the use of stem cells, including DSCs, to cure Alzheimer’s disease symptoms [54, 55]. In 2017, Wang et al. reported regeneration of damaged neurons cocultured with DPSCs [56]. This observation further revealed enhanced viability and impedance of apoptosis in neuroblastoma cells. Similarly, when cocultured with primary hippocampal and ventral mesencephalic neurons, DPSCs showed exceptional protection against the β-amyloid protein, indicating a neuroprotective activity in Alzheimer’s disease [57]. The DPSCs expressed the neuronal phenotype and produced neurotrophic factors to rescue primary neurons. Similarly, in another study [54], when SHEDs were transplanted in a mouse model of Alzheimer’s disease, it engendered substantial cognitive function improvement attributable to multiple factors, such as neuroprotection, axonal elongation, neurotransmission, reduced inflammation, and microglial regulation.

### Parkinson’s disease

Parkinson’s disease, a neurodegenerative disease, is characterized by the progressive death of substantia nigra dopaminergic neurons, resulting in a regional loss of striatal dopamine. Accumulating evidence indicates that the DSCs provide therapeutic possibilities in Parkinson’s disease. In 2011, Nesti et al. studied the neuroprotective effects of DPSC against 1-methyl-4-phenylpyridinium (MPP^+^) and rotenone using an indirect coculture system with mesencephalic cell cultures [58]. They found that the coculture significantly attenuated MPP^+^- or rotenone-induced toxicity in the dopaminergic neuron. Moreover, the conditioned medium derived from these cells protected primary neurons from 6-hydroxydopamine (6-OHDA)-induced toxicity and enhanced neurite outgrowth [34]. Similarly, through this attenuation of 6-OHDA-induced toxicity and improved cell viability, DPSCs protected the primary neurons [57]. The neuroprotective potential of exosomes derived from SHEDs on human dopaminergic neurons revealed that the exosomes suppress 6-OHDA-induced apoptosis in dopaminergic neurons [34]. In-vivo results corroborated the in-vitro results. In a rat model of Parkinson’s disease, the transplantation of dopaminergic neuron-like cells from SHEDs reduced the 6-OHDA-induced neurodegeneration [59]. Similarly, Parkinsonian rats achieved neurological performance after SHED sphere transplantation; the sphere engraftment improved the apomorphine-evoked rotation of behavioral disorders in rats [60].

### Spinal cord injury

SCI is a debilitating neurological disorder posing severe clinical and socioeconomic burden. SCI, coupled with a range of complex and long-term sequelae, considerably reduces the quality of life of the affected individual. In this context, DSC-based transplantation strategies hold great potential. Dental pulp cells grafted in rat hemisected spinal cord could promote motor neuron survival [20]. DPSC transplantation in a completely transected spinal cord considerably improved hindlimb locomotor functions, accompanied by improved preservation of neural elements [61]. Moreover, the transplantation of human DPSCs along with chitosan scaffolds into an SCI rat model showed substantial spontaneous functional recovery of the hindlimb [52]. Similarly, SHED transplantation in SCI rodents resulted in considerable improvement in the behavioral outcome; this improvement was attributable to a reduction of the cystic cavity and glial scar and to the enhancement of neurofilament density near the lesion site [62]. Furthermore, in 2012 Taghipour et al. indicated that the transplantation of undifferentiated and differentiated SHEDs promoted functional recovery in a rat spinal cord contusion injury model by differentiating into the cells of the neuronal lineage [63].

### Stroke

Stroke triggers a cascade of events leading to the loss of a large variety of neural cells and secondary neurodegeneration, in many cases leading to permanent disability. Thus, the primary challenge in stroke therapeutics is to improve functional recovery at the organismal, cellular, and molecular levels. Many recent studies have applied cell therapy in stroke recovery models. Our group has developed several such therapies with limited success in aged animal models of stroke [64–67].

Stem cell-based therapies for stroke use different cell sources. Therapeutic translational studies using DPSCs for stroke treatment in a cerebral ischemic rodent model have provided promising results. Transplantation of a porcine CD31^−^/CD146^−^ side population (SP) of dental pulp cells accelerated neovascularization of the ischemic zone and enhanced neuronal regeneration [68]. Furthermore, the intracerebral transplantation of human DPSCs after focal cerebral ischemia in a rodent model considerably improved forelimb sensory motor function [69]. Identical outcomes were observed after DPSC delivery in permanent middle cerebral artery occlusion (MCAO) rats, where the grafted cells shrunk the peri-infarct lesion and enhanced functional recovery [21]. Similarly, SHED engraftment into a hypoxic–ischemic injured brain resulted in remarkable neurological and pathophysiological improvement [70]. Intranasal administration of conditioned media derived from SHEDs (SHED-CM) in a MCAO model promoted vasculogenesis and endogenous neural progenitor cell (NPC) migration, as well as differentiation and amelioration of ischemic brain injury [19].

### PNS diseases

The neural crest origin of DSCs makes them a perfect candidate for cell therapy of PNS disorders. Recent findings indicate that DPSCs ameliorate diabetic polyneuropathy by increasing impaired sciatic nerve blood flow, sciatic motor-sensory nerve conduction velocity, and capillary number-to-muscle and intra-epidermal nerve fiber density ratio [71]. When isolated from patients with neurofibromatosis type 1, DPSCs have a proliferation rate higher than that of normal cells; thus, DPSCs represent a suitable model for neurofibromatosis type 1 [72]. Moreover, DPSC-derived oligoprogenitor cells showed high therapeutic potential in an animal model of sciatic nerve injury [73], indicating its potential as a therapeutic for amelioration of myelin injuries in the PNS [74]. When SHED-CM was investigated for peripheral nerve regeneration, SHED-CM-treated Schwann cells exhibited a significantly increased number of neuronal and angiogenesis related genes.

In addition, SHED-CM stimulated neuritogenesis of dorsal root ganglia and increased cell viability [75]. Furthermore, poly(ε-caprolactone)/gelatin nanofibrous nerve guide seeded with DSCs for peripheral nerve regeneration were transplanted at the site of nerve injury and resulted in nerve survival and axonal regeneration in rat sciatic nerves [76]. More recently, we assessed the potential of three-dimensional printing in improving long-distance nerve guide regeneration strategies [77].

## Potential biomechanism underlying DSC-mediated functional recovery

With the failure of neuroprotective strategies in salvaging or replacing injured CNS tissues, the focus on neurorestorative therapies has increased [78]. Neurorestorative treatments encompass the delivery of exogenous stem cells or recruitment of endogenous cells [79]. In general, when exogenous stem cells are used, the transplanted cells may engraft, differentiate, and finally integrate into the damaged CNS, thus replacing the lost neural cells [8]. In addition to cell replacement, numerous studies have investigated the mechanisms that contribute to the recovery; these are summarized below.

### Cell replacement

Understanding how DSC-based therapy may improve function in neurological disorders requires further research, although replacement has always been proposed as a primary salvage mechanism. In the above context, and because of their pluripotent nature, DSCs are widely accepted as a choice for transplantation since they differentiate and integrate into the recipient tissue post-transplantation [22, 51, 80]. Substantial evidence indicates that, after transplantation, DSCs differentiate into several neuronal cell types such as GABAergic, glutamatergic [81], dopaminergic [60], neuronal, glial [82], and Schwann [83] cells. Notably, the engrafted cells exhibit tetradoxin-sensitive voltage-dependent sodium currents and tetraethyl ammonium-sensitive delayed rectifier potassium currents [84, 85], suggesting the retention of electrophysiological characteristics by these cells. When transplanted, the DPSCs express the early neuronal marker N-tubulin, the neuronal-specific intermediate filament protein NF-M, the postmitotic neuronal marker NeuN, and glial fibrillary acidic protein (GFAP), indicating a population similar to neuronal satellite cells [86]. Similarly, quantitative analysis of undifferentiated and differentiated SHEDs after 5 weeks of transplantation shows expression of microtubule-associated protein 2 (MAP2), neural cell adhesion molecule (NCAM), and nestin; also seen are a few Ki67-positive cells, the myelin basic protein marker S100, neural/glial antigen 2 (NG2), and the astrocyte marker GFAP. A significant functional recovery was achieved which corroborated well with the SHED integration [63], thus indicating that these cells can be a suitable candidate for neurodegenerative disease recovery. Likewise, when transplanted into a completely transected spinal cord, approximately 90% of the engrafted SHEDs differentiated into mature oligodendrocytes, expressing antigen-presenting cells and the myelin basic protein [20], again suggesting the beneficial effect of autonomous cell activities.

Cell replacement can also be achieved by inducing endogenous stem cells to migrate to the diseased or injured area. When transplanted into mice hippocampus, DPSCs influence the recruitment of endogenous neural stem cells [44]. The long-term transplantation effect indicates that newly produced neurons undergo proliferation to form NPCs and neurons at the graft site. SHED-CM could promote the migration of NPCs as per the quantification of doublecortin (DCX)-positive neurons. In addition to replacing lost neurons through promoting migration, SHED-CM can also promote differentiation of endogenous NPCs in the ischemic brain [19]. A few studies reported the ability of the exogenous DSCs to stimulate endogenous neurogenesis [19, 44, 87], reinforcing the possibility of exploiting the process of adult neurogenesis, and enhancing the neurogenic capacity of DSC.

### Paracrine effect

A recent paradigm shift has suggested that the beneficial effects of stem cells [84], including DSCs, are at least in part due to their paracrine actions. A stem cell-mediated paracrine (or bystander) effect is a method of communication in which trophic factors secreted by the implanted cells modulate the molecular composition of the environment and evoke responses from resident cells. The trophic factors released by the stem cells are responsible for the development, maintenance, repair, and survival of the neuronal population [88–92].

In animal experiments, DSCs provide cytoprotection through secretion of neurotropic peptides, which contribute to neural repair and regeneration [26, 91, 93–96]. The tissue concentrations of vascular endothelial growth factor (VEGF), nerve growth factor (NGF), brain-derived neurotrophic factor (BDNF), glial cell-derived neurotrophic factor (GDNF), ciliary neurotrophic factor, and neurotrophin-3 (NT3) were significantly increased after DSC transplantation in various neurological disorders [20, 26, 60, 97], indicating that DCS-mediated improvement is at least partly contributed to via neurotrophin secretion. Nosrat et al. showed that, when DPSCs interacted in vivo with the developing host nervous system, neuroplastic changes were observed which were attributed to the chemicals secreted by the DPSCs. The authors illustrated that this chemoattraction of avian trigeminal ganglion axons toward implanted DPSCs was mediated by stromal cell-derived factor-1 (SDF1) and its receptor, C-X-C chemokine receptor type 4 (CXCR4) [98]. DPSCs when cocultured with trigeminal neurons promoted the survival of trigeminal neurons and elaborated neurite outgrowth by secreting growth factors such as NGF, BDNF, and GDNF. Furthermore, when transplanted, cells ectopically innervated into the anterior chamber of the eye of rats, indicating that DSCs produced neurotrophic factors during development [98]. When DPSCs or SHEDs were grafted in a SCI rodent model, high expression of neurotrophic soluble factors was observed [20] which increased the number of surviving motor neurons [98], signifying a functional bioactivity of the DSC-derived neurotrophic factors in vivo. Furthermore, SHED transplantation caused considerable neurological and pathophysiological recovery in neonatal mice; however, after 8 weeks of transplantation, no new neurons, oligodendrocytes, or astrocytes were obtained, indicating that the improvement achieved was through non-neural replacement mechanisms [70]. Taken together, the aforementioned results suggest that both DPSCs and SHEDs are a promising cell therapy source to understand neurotrophic factor-mediated neurorestoration.

### Vasculogenesis

Despite having limited self-repair abilities [78], the CNS can achieve some degree of spontaneous recovery. A promising field of investigation has focused on triggering and stimulating the CNS self-repair system to regenerate new neurons [79] or establish an effective vascular network [99]. The formation of new vessels is a complex process involving various growth factors, chemokines, and mural cells (i.e., the cells involved in normal vasculature formation), all of which play different roles in promoting and refining this process [99]. DSCs are considered to establish therapeutic angiogenesis either through differentiation into vascular cells (e.g., endothelial cells) or through paracrine angiogenic growth factor secretion [20, 100].

The dental pulp tissue is a highly innervated and vascularized tissue; in other words, it contains blood vessels and neuron precursor cells. Thus, DPSCs can differentiate into vascular and neuronal cells [101]. DPSCs release angiogenic factors and cytokines, such as VEGF, SDF, monocyte chemotactic protein 1 (MCP1; chemokine C-C motif ligand 2), and platelet-derived growth factor (PDGF)BB [102]. The trophic factors expressed by stem cells are critical for vascular network remodeling; for instance, VEGF may be crucial in DSC-mediated vascular repair [20, 53, 103] because it may facilitate DSCs to bypass the blood–brain barrier (BBB) [104]. DPSCs mediate localized discontinuities in the BBB by upregulating VEGFα expression, enabling their passage into the brain parenchyma. Similarly, the transplantation of a dental pulp side population (SP) is essential when the transplanted blood flow to the infarcted brain increases through enhanced expression of VEGF [105]. Furthermore, SHED-CM induces vasculogenesis in ischemic brain injury after permanent MCAO, as revealed by high staining of endothelial cell antigen in the peri-infarct area [20], thus indicating the association of growth factors with vascularization. Moreover, SHED-CM-treated Schwann cells exhibited significantly increased proliferation, migration, and expression of neurons, the extracellular matrix, and angiogenesis-related genes in a rat sciatic nerve injury model. The concentration of VEGF was found elevated in SHED-CM [75].

Notably, in 2015, Shen et al. [100] showed that DPSC-conditioned media can induce migration and tube formation in vascular smooth muscle cells and human umbilical vein endothelial cells, suggesting that DPSCs can produce vessel-like structures. Thus, it is reasonable to hypothesize that both DPSCs and SHEDs have vasculogenic differentiation potential, and can enhance angiogenesis through various modes of action.

### Synaptogenesis

Studies demonstrating the synaptogenic potential of DSCs, either in vitro or in vivo, are rare; the first study reporting neuroplastic changes in DPSCs was obtained using an avian embryonic model system where engrafted DPSCs secreted neurotrophic factors which coordinated axon guidance within the recipient host nervous system [91]. The secreted neurotrophic factors were responsible for maintaining the integrity and plasticity of neuronal circuits through a process involving competition between the synapses of different axons [106]. Similarly, enhanced neuroplasticity was observed when human DPSCs were transplanted in ischemic [107] and hypoxic–ischemic [108] brains. The insulin growth factor receptor 1/insulin growth factor 1 [107, 109] and CXCR4/SDF1α [107] signaling pathways, known to modulate normal dendritic growth and synapse formation, were found to be associated with the observed plasticity, as evident through neurite regeneration [107, 110]. Furthermore, both human DPSCs and SHEDs modulate synaptogenesis through the Sonic hedgehog (SHH) signaling pathway [111], a pathway with a well-documented role in synaptogenesis [112]. The gene ontology analysis of DPSCs, PDLSC, and ABMSCs suggests that these cells possess a plasticity nature [113]. Thus, DSCs may induce functional recovery by modulating the synaptogenic mechanism.

### Immunomodulation

In addition to lost neuron substitution, immunomodulation is a potential neurorestorative tool. The immune system is crucial in cell replacement. If the interaction between the transplant and the immune system is not considered, the implant may be rejected by the body, leading to detrimental clinical consequences. Recently, the immunomodulatory potential of DSCs has been explored. Accumulating evidence indicates that DSCs affect innate and adaptive immune cells through two possible mechanisms: direct cell–cell contact, and the release of various soluble factors. This section focuses on both paths through which immunorestoration can be achieved.

The interaction between DSCs and immune cell types revealed that DSCs provide protection by downregulating T cells [103] and B cells [114, 115] and increasing resistance to natural killer (NK) cells [114]. This interaction may modulate the expression of transduction signaling mechanisms, thus augmenting the inhibition of lymphocyte and NK cell production; for instance, when SHEDs were transplanted into an experimental autoimmune encephalomyelitis model, they inhibited the immune response by suppressing T cells and inducing regulatory T cells (Tregs) [33]. SHEDs can also induce the immunoregulatory phenotype in monocyte-derived dendritic cells and macrophages [33]. The aforementioned immunomodulator activities indicate that SHEDs could be suitable for suppressing graft-versus-host reactions and treating neuronal autoimmune disorders of the CNS.

DSCs modulate immunological responses by secreting a complex set of trophic factors that significantly contribute to injury repair [66, 92, 116, 117]. DPSCs inhibit stimulated T-cell proliferation, most likely through transforming growth factor (TGF)-β1 and interleukin (IL)-10 signaling [118]. This study illustrated that, when CD4^+^ T cells were cocultured with DPSCs, the T cells demonstrated a high Treg expression. However, blocking TGF-β1 and IL-10 signaling resulted in a low Treg count, indicating that DPSCs require stimulatory factors to exert their effects. Similarly, SHEDs can nullify the proinflammatory effects by downregulating the expression of proinflammatory cytokines (e.g., IL-1β and tumor necrosis factor (TNF)-α) and upregulating that of anti-inflammatory cytokines (e.g., IL-4 and IL-10) [70]. Most of these cytokines are involved in reactive astrogliosis, a process that might contribute to protection [119–123]. Furthermore, SHEDs can change the polarity of microglia or macrophages from M1 to M2 to suppress proinflammatory mediators and enhance tissue repair. M2-like microglia or macrophages are cells responsible for promoting tissue repair through various pathways, including anti-inflammatory cytokine secretion [124], cellular debris phagocytosis [125], axonal elongation [126], and proliferation and differentiation of oligodendrocyte progenitor cells [127]. Thus, it is reasonable to say that DSCs exert immunorestoration through various mechanisms, and their immunosuppressive potential provides a distinctive advantage for the clinical management of neurodegenerative disorders.

### Apoptosis

One of the aims of stem cell therapy is to suppress apoptosis to prevent early secondary cell death. Apoptosis accounts for approximately 90% of neuronal loss in CNS injury models [128, 129], making it an important avenue for treatment. Both SHEDs [130] and DPSCs [20] can reduce cell loss through apoptosis attenuation, thus contributing to tissue and motor neuron preservation. When SHEDs were transplanted in an SCI model, they prevented early apoptosis [130]. Likewise, SHED-derived exosomes and SHED-CM improved the neurological outcome by inhibiting apoptosis in an in-vitro dopaminergic neuronal model [34] and in-vivo hypoxic–ischemic model [70], respectively, as revealed by the positive expression of effector caspases 3 and 7 in both cases [34]. The ability of DSCs to secrete cytokines, such as VEGF and MCP1, can also contribute to the restorative process, as these cytokines can neutralize the effects of apoptosis [102, 131]. For example, VEGF is instrumental in preventing serum starvation-induced apoptosis by upregulating B-cell lymphoma 2 (Bcl-2) expression in vascular endothelial cells [132]. Similarly, DPSCs significantly reduce the cytotoxicity of β-amyloid peptide by stimulating the activity of the endogenous survival factor Bcl-2 and reducing that of the apoptotic regulator Bcl-2-associated X protein (Bax) [133]. To prevent apoptosis, DPSCs secrete classic apoptosis inhibitor proteins such as Bcl-2 [133] and downregulate the expression of the apoptotic regulator Bax. The Bcl-2/Bax ratio is critical for the cells to obtain a pathological stimulus [134]. High Bcl-2 expression prevents caspase inhibitor release, making the neuronal cells less likely to respond to apoptotic signaling [135]. Taken together, DPSCs and SHEDS may achieve restoration by preventing apoptosis, and DSCs may have therapeutic potential specifically as a stimulator and modulator of the local repair response in the CNS.

## Conclusion

DSCs are being explored as a new cell source for cell therapy in neurodegenerative diseases. Due to their accessibility, plasticity, and ethical suitability they have become an attractive source of ready-to-use autologous transplantation cells in neurological disorders. However, a comprehensive understanding of the healing processes in the CNS tissue triggered by DSC-based therapies has not yet been achieved. Recent advancements in cell therapy technologies have revealed that these cells provide benefits through multiple mechanisms: cell integration, a bystander effect, vasculogenesis, immunomodulation, and by inhibiting apoptosis. Numerous cellular and preclinical studies have indicated the role of each of these mechanisms in achieving neurological recovery. However, many of these effects of DSCs have drawbacks; for example, transdifferentiation seems to occur at too low a frequency to account for the meaningful improvement. Furthermore, the amount of secreted neurotropins does not allow to exert an effect on the nearby vicinity. In addition it is not clear to what extent the above discussed mechanisms contribute to the functional recovery. There needs to be further elucidation of the fundamental biological mechanisms responsible for molecular and functional recovery. To conclude, it is reasonable that DSC-mediated neurorestorative therapy has a promising future with applications in neural tissue regeneration and neurological disorder management.

## Abbreviations

6-OHDA: 6-Hydroxydopamine
ABMSC: Alveolar bone-derived mesenchymal stem cell
Bax: B-cell lymphoma 2-associated X protein
BBB: Blood–brain barrier
Bcl-2: B-cell lymphoma 2
BDNF: Brain-derived neurotrophic factor
BMMSC: Bone marrow-derived mesenchymal stem cell
CNS: Central nervous system
CXCR4: C-X-C chemokine receptor type 4
DCX: Doublecortin
DPSC: Dental pulp stem cell
DSC: Dental-derived stem cell
GDNF: Glial cell-derived neurotrophic factor
GFAP: Glial fibrillary acidic protein
IL: Interleukin
MAP 2: Microtubule-associated protein 2
MCAO: Middle cerebral artery occlusion
MCP1: Monocyte chemoattractant protein 1
MPP: 1-Methyl-4-phenylpyridinium
MSC: Mesenchymal stem cell
NCAM: Neural cell adhesion molecule
NG2: Neural/glial antigen 2
NGF: Nerve growth factor
NK: Natural killer
NPC: Neural progenitor cell
NSC: Neural stem cell
NT3: Neurotrophin-3
PDGF: Platelet-derived growth factor
PDLSC: Periodontal ligament stem cell
PNS: Peripheral nervous system
SCI: Spinal cord injury
SDF1: Stromal cell-derived factor 1
SHED: Stem cell from human exfoliated deciduous teeth
SHED-CM: Stem cell from human exfoliated deciduous teeth-conditioned media
SHH: Sonic hedgehog
SP: Side population
TGF: Transforming growth factor
TNF: Tumor necrosis factor
Treg: Regulatory T cell
VEGF: Vascular endothelial growth factor

## References

[1] Uryu K, Haddix T, Robinson J, Nakashima-Yasuda H, Lee VM, Trojanowski JQ (2010). Burden of neurodegenerative diseases in a cohort of medical examiner subjects. J Forensic Sci.

[2] Sanai N, Tramontin AD, Quinones-Hinojosa A, Barbaro NM, Gupta N, Kunwar S, Lawton MT, McDermott MW, Parsa AT, Manuel-Garcıa Verdugo J, Berger MS, Alvarez-Buylla A (2004). Unique astrocyte ribbon in adult human brain contains neural stem cells but lacks chain migration. Nature.

[3] Arsenijevic Y, Villemure JG, Brunet JF, Bloch JJ, De’Glon N, Kostic C, Zurn A, Aebischer P (2001). Isolation of multipotent neural precursors residing in the cortex of the adult human brain. Exp Neurol.

[4] Roy NS, Wang S, Jiang L, Kang J, Benraiss A, Harrison-Restelli C, Fraser RA, Couldwell WT, Kawaguchi A, Okano H, Nedergaard M, Goldman SA (2000). In-vitro neurogenesis by progenitor cells isolated from the adult human hippocampus. Nat Med.

[5] Eriksson PS, Perfilieva E, Bjork-Eriksson T, Alborn AM, Nordborg C, Peterson DA, Gage FH (1998). Neurogenesis in the adult human hippocampus. Nat Med.

[6] Goldman SA (2016). Stem and progenitor cell-based therapy of the central nervous system: hopes, hype and wishful thinking. Cell Stem Cell.

[7] Hess DC, Borlongan CV (2008). Stem cells and neurological diseases. Cell Prolif.

[8] Chopp M, Li Y (2002). Treatment of neural injury with marrow stromal cells. Lancet Neurol.

[9] Caplan AI, Bruder SP (2001). Mesenchymal stem cells: building blocks for molecular medicine in the 21st century. Trends Mol Med.

[10] Gronthos S, Brahim J, Li W, Fisher LW, Cherman N, Boyde A, DenBesten P, Robey PG, Shi S (2002). Stem cell properties of human dental pulp stem cells. J Dent Res.

[11] Miura M, Gronthos S, Zhao M, Lu B, Fisher LW, Robey PG, Shi S (2003). SHED: stem cells from human exfoliated deciduous teeth. Proc NatlAcadSci USA.

[12] Sonoyama W, Liu Y, Fang D, Yamaza T, Seo BM, Zhang C, Liu H, Gronthos S, Wang CY, Shi S, Wang S (2006). Mesenchymal stem cell-mediated functional tooth regeneration in swine. PLoS One.

[13] Ikeda E, Yagi K, Kojima M, Yagyuu T, Ohshima A, Sobajima S, Tadokoro M, Katsube Y, Isoda K, Kondoh M, Kawase M, Go MJ, Adachi H, Yokota Y, Kirita T, Ohgushi H (2008). Multipotent cells from the human third molar: feasibility of cell-based therapy for liver disease. Differentiation.

[14] Zhang Q, Shi S, Liu Y, Uyanne J, Shi Y, Shi S, Le AD (2009). Mesenchymal stem cells derived from human gingiva are capable of immunomodulatory functions and ameliorate inflammation-related tissue destruction in experimental colitis. J Immunol.

[15] Morsczeck C, Gotz W, Schierholz J, Zeilhofer F, Kuhn U, Mohl C, Sippel C, Hoffmann KH (2005). Isolation of precursor cells (PCs) from human dental follicle of wisdom teeth. Matrix Biol.

[16] Matsubara T, Suardita K, Ishii M, Sugiyama M, Igarashi A, Oda R, Nishimura M, Saito M, Nakagawa K, Yamanaka K, Miyazaki K, Shimizu M, Bhawal UK, Tsuji K, Nakamura K, Kato Y (2005). Alveolar bone marrow as a cell source for regenerative medicine: differences between alveolar and iliac bone marrow stromal cells. J Bone Miner Res.

[17] Seo BM, Miura M, Gronthos S, Bartold PM, Batouli S, Brahim J, Young M, Robey PG, Wang CY, Shi S (2004). Investigation of multipotent postnatal stem cells from human periodontal ligament. Lancet.

[18] Chun SY, Soker S, Jang YJ, Kwon TG, Yoo ES (2016). Differentiation of human dental pulp stem cells into dopaminergic neuron-like cells in vitro. Korean Med Sci.

[19] Inoue T, Sugiyama M, Hattori H, Wakita H, Wakabayashi T, Ueda M (2013). Stem cells from human exfoliated deciduous tooth-derived conditioned medium enhance recovery of focal cerebral ischemia in rats. Tissue Eng Part A.

[20] Sakai K, Yamamoto A, Matsubara K, Nakamura S, Naruse M, Yamagata M, Sakamoto K, Tauchi R, Wakao N, Imagama S, Hibi H, Kadomatsu K, Ishiguro N, Ueda M (2012). Human dental pulp-derived stem cells promote locomotor recovery after complete transection of the rat spinal cord by multiple neuroregenerative mechanisms. J Clin Invest.

[21] Leong WK, Henshall TL, Arthur A, Kremer KL, Lewis MD, Helps SC, Field J, Hamilton-Bruce MA, Warming S, Manavis J, Vink R, Gronthos S, Koblar SA (2012). Human adult dental pulp stem cells enhance post-stroke functional recovery through non-neural replacement mechanisms. Stem Cells Transl Med.

[22] Kiraly M, Kadar K, Horvathy DB (2011). Integration of neuronally pre-differentiated human dental pulp stem cells into rat brain in vivo. Neurochem Int.

[23] Lee HS, Jeon M, Kim SO, Kim SH, Lee JH, Ahn SJ, Shin Y, Song JS (2015). Characteristics of stem cells from human exfoliated deciduous teeth (SHED) from intact cryopreserved deciduous teeth. Cryobiology.

[24] Ishiy FA, Fanganiello RD, Griesi-Oliveira K, Suzuki AM, Kobayashi GS, Morales AG, Capelo LP, Passos-Bueno MR (2015). Improvement of in vitro osteogenic potential through differentiation of induced pluripotent stem cells from human exfoliated dental tissue towards mesenchymal-like stem cells. Stem Cells Int.

[25] Li D, Deng T, Li H, Li Y (2015). MiR-143 and miR-135 inhibitors treatment induces skeletal myogenic differentiation of human adult dental pulp stem cells. Arch Oral Biol.

[26] Chang CC, Chang KC, Tsai SJ, Chang HH, Lin CP (2014). Neurogenic differentiation of dental pulp stem cells to neuron-like cells in dopaminergic and motor neuronal inductive media. J Formos Med Assoc.

[27] Jang Soomi, Kang Young-Hoon, Ullah Imran, Shivakumar Sharath, Rho Gyu-Jin, Cho Yeong-Cheol, Sung Iel-Yong, Park Bong-Wook (2018). Cholinergic Nerve Differentiation of Mesenchymal Stem Cells Derived from Long-Term Cryopreserved Human Dental Pulp In Vitro and Analysis of Their Motor Nerve Regeneration Potential In Vivo. International Journal of Molecular Sciences.

[28] Gnanasegaran N, Govindasamy V, Mani V, Abu Kasim NH (2017). Neuroimmunomodulatory properties of DPSCs in an in vitro model of Parkinson's disease. IUBMB Life.

[29] Mortada I, Mortada R, Al Bazzal M. Dental pulp stem cells and neurogenesis. Adv Exp Med Biol. 2017. 10.1007/5584_2017_71 28687960

[30] Urraca N, Memon R, El-Iyachi I, Goorha S, Valdez C, Tran QT (2015). Characterization of neurons from immortalized dental pulp stem cells for the study of neurogenetic disorders. Stem Cell Res.

[31] Liang D, Chang RJ, Chinb AJ, Smitha A, Kelly C, Weinberg ES, Ge R (2001). The role of vascular endothelial growth factor (VEGF) in vasculogenesis, angiogenesis and hematopoiesis in zebrafish development. Mech Dev.

[32] Bae YC, Paik SK, Park KP, Ma SK, Jin JG, Ahn DK, Kim SK, Moritani M, Yoshida A (2004). Quantitative analysis of tooth pulp afferent terminals in the rat brain stem. Neuroreport.

[33] Rossato C, Brandao WN, Castro SBR, de Almeida DC, Maranduba CMC, Camara NOS, Peron JPS, Silva FS (2017). Stem cells from human exfoliated deciduous teeth reduce tissue- infiltrating inflammatory cells improving clinical signs in experimental autoimmune encephalomyelitis. Biologicals.

[34] Jarmalaviciute A, Tunaitis V, Pivoraite U, Venalis A, Pivoriunas A (2015). Exosomes from dental pulp stem cells rescue human dopaminergic neurons from 6-hydroxydopamine-induced apoptosis. Cytotherapy.

[35] Yalvac ME, Rizvanov AA, Kilic E, Sahin F, Mukhamedyarov MA, Islamov RR, Palotas A (2009). Potential role of dental stem cells in the cellular therapy of cerebral ischemia. Curr Pharm Des.

[36] Kerkis I, Ambrosio CE, Kerkis A, Martins DS, Zucconi E, Fonseca SA, Cabral RM, Maranduba CM, Gaiad TP, Morini AC, Vieira NM, Brolio MP, Sant'Anna OA, Miglino MA, Zatz M (2008). Early transplantation of human immature dental pulp stem cells from baby teeth to golden retriever muscular dystrophy (GRMD) dogs: local or systemic?. J Transl Med.

[37] Alipour R, Adib M, Masoumi Karimi M, Hashemi-Beni B, Sereshki N (2013). Comparing the immunoregulatory effects of stem cells from human exfoliated deciduous teeth and bone marrow-derived mesenchymal stem cells. Iran J Allergy Asthma Immunol.

[38] Komada Yukiya, Yamane Toshiyuki, Kadota Daiji, Isono Kana, Takakura Nobuyuki, Hayashi Shin-Ichi, Yamazaki Hidetoshi (2012). Origins and Properties of Dental, Thymic, and Bone Marrow Mesenchymal Cells and Their Stem Cells. PLoS ONE.

[39] Gazarian KG, Ramírez-García LR (2017). Human deciduous teeth stem cells (SHED) display neural crest signature characters. PLoS One.

[40] Akiyama K, Chen C, Gronthos S, Shi S (2012). Lineage differentiation of mesenchymal stem cells from dental pulp, apical papilla, and periodontal ligament. Methods Mol Biol.

[41] Shi S, Bartold PM, Miura M, Seo BM, Robey PG, Gronthos S (2005). The efficacy of mesenchymal stem cells to regenerate and repair dental structures. Orthod Cranio fac Res.

[42] Wilson R, Urraca N, Skobowiat C, Hope KA, Miravalle L, Chamberlin R, Donaldson M, Seagroves TN, Reiter LT (2015). Assessment of the tumorigenic potential of spontaneously immortalized and hTERT-immortalized cultured dental pulp stem cells. Stem Cells Transl Med.

[43] Isobe Y, Koyama N, Nakao K, Osawa K, Ikeno M, Yamanaka S, Okubo Y, Fujimura K, Bessho K (2016). Comparison of human mesenchymal stem cells derived from bone marrow, synovial fluid, adult dental pulp and exfoliated deciduous tooth pulp. Int J Oral Maxillo fac Surg.

[44] Huang AH, Snyder BR, Cheng PH, Chan AW (2008). Putative dental pulp-derived stem/stromal cells promote proliferation and differentiation of endogenous neural cells in the hippocampus of mice. Stem Cells.

[45] Karbalaie K, Tanhaei S, Rabiei F, Kiani-Esfahani A, Masoudi NS, Nasr-Esfahani MH, Baharvand H (2015). Stem cells from human exfoliated deciduous tooth exhibit stromal-derived inducing activity and lead to generation of neural crest cells from human embryonic stem cells. Cell J.

[46] Karaoz E, Demircan PC, Saglam O, Aksoy A, Kaymaz F, Duruksu G (2011). Human dental pulp stem cells demonstrate better neural and epithelial stem cell properties than bone marrow-derived mesenchymal stem cells. Histochem Cell Biol.

[47] Ren H, Sang Y, Zhang F, Liu Z, Qi N, Chen Y (2016). Comparative analysis of human mesenchymal stem cells from umbilical cord, dental pulp and menstrual blood as sources for cell therapy. Stem Cells Int.

[48] Kang CM, Kim H, Song JS, Choi BJ, Kim SO, Jung HS, Moon SJ, Choi HJ (2016). Genetic comparison of stemness of human umbilical cord and dental pulp. Stem Cells Int.

[49] Huang GT, Gronthos S, Shi S (2009). Mesenchymal stem cells derived from dental tissues vs. those from other sources: their biology and role in regenerative medicine. J Dent Res.

[50] Gervois P, Wolfs E, Dillen Y, Hilkens P, Ratajczak J, Driesen RB, Vangansewinkel T, Bronckaers A, Brone B, Struys T, Lambrichts I (2017). Paracrine maturation and migration of SH-SY5Y cells by dental pulp stem cells. J Dent Res.

[51] Song M, Lee JH, Bae J, Bu Y, Kim EC (2017). Human dental pulp stem cells are more effective than human bone marrow-derived mesenchymal stem cells in cerebral ischemic injury. Cell Transplant.

[52] Zhang J, Lu X, Feng G, Gu Z, Sun Y, Bao G, Xu G, Lu Y, Chen J, Xu L, Feng X, Cui Z (2016). Chitosan scaffolds induce human dental pulp stem cells to neural differentiation: potential roles for spinal cord injury therapy. Cell Tissue Res.

[53] Arthur A, Rychkov G, Shi S, Koblar SA, Gronthos S (2008). Adult human dental pulp stem cells differentiate toward functionally active neurons under appropriate environmental cues. Stem Cells.

[54] Mita T, Furukawa-Hibi Y, Takeuchi H, Hattori H, Yamada K, Hibi H, Ueda M, Yamamoto A (2015). Conditioned medium from the stem cells of human dental pulp improves cognitive function in a mouse model of Alzheimer's disease. Behav Brain Res.

[55] Wang SS, Jia J, Wang Z (2018). Mesenchymal stem cell-derived extracellular vesicles suppresses iNOS expression and ameliorates neural impairment in Alzheimer's disease mice. J Alzheimers Dis.

[56] Wang F, Jia Y, Liu J, Zhai J, Cao N, Yue W, He H, Pei X (2017). Dental pulp stem cells promote regeneration of damaged neuron cells on the cellular model of Alzheimer's disease. Cell Biol Int.

[57] Apel C, Forlenza OV, de Paula VJ, Talib LL, Denecke B, Eduardo CP (2009). The neuroprotective effect of dental pulp cells in models of Alzheimer's and Parkinson's disease. J Neural Transm.

[58] Nesti C, Pardini C, Barachini SD, Alessandro D, Siciliano G, Murri L, Petrini M, Vaglini F (2011). Human dental pulp stem cells protect mouse dopaminergic neurons against MPP^+^ or rotenone. Brain Res.

[59] Wang J, Wang X, Sun Z, Wang X, Yang H, Shi S, Wang S (2010). Stem cells from human exfoliated deciduous teeth can differentiate into dopaminergic neuron-like cells. Stem Cells Dev.

[60] Fujii H, Matsubara K, Sakai K, Ito M, Ohno K, Ueda M, Yamamoto A (2015). Dopaminergic differentiation of stem cells from human deciduous teeth and their therapeutic benefits for parkinsonian rats. Brain Res.

[61] Yamamoto A, Sakai K, Matsubara K, Kano F, Ueda M (2014). Multifaceted neuro-regenerative activities of human dental pulp stem cells for functional recovery after spinal cord injury. Neurosci Res.

[62] Nicola FC, Rodrigues LP, Crestani T, Quintiliano K, Sanches EF, Willborn S, Aristimunha D, Boisserand L, Pranke P, Netto CA (2016). Human dental pulp stem cells transplantation combined with treadmill training in rats after traumatic spinal cord injury. Braz J Med Biol Res.

[63] Taghipour Z, Karbalaie K, Kiani A, Niapour A, Bahramian H, Nasr-Esfahani MH, Baharvand H (2012). Transplantation of undifferentiated and induced human exfoliated deciduous teeth-derived stem cells promote functional recovery of rat spinal cord contusion injury model. Stem Cells Dev.

[64] Tatarishvili J, Oki K, Monni E, Koch P, Memanishvili T (2014). Human induced pluripotent stem cells improve recovery in stroke-injured aged rats. Restor Neurol Neurosci.

[65] Balseanu AT, Buga AM, Catalin B, Wagner DC, Boltze J, Zagrean AM (2014). Multimodal approaches for regenerative stroke therapies: combination of granulocyte colony-stimulating factor with bone marrow mesenchymal stem cells is not superior to G-CSF alone. Front Aging Neurosci.

[66] Popa-Wagner A, Buga AM, Doeppner TR, Hermann DM (2014). Stem cell therapies in preclinical models of stroke associated with aging. Front Cell Neurosci.

[67] Buga AM, Scheibe J, Moller K, Ciobanu O, Posel C (2015). Granulocyte colony-stimulating factor and bone marrow mononuclear cells for stroke treatment in the aged brain. Curr Neurovasc Res.

[68] Sugiyama M, Iohara K, Wakita H, Hattori H, Ueda M, Matsushita K, Nakashima M (2011). Dental pulp-derived CD31(−)/CD146(−) side population stem/progenitor cells enhance recovery of focal cerebral ischemia in rats. Tissue Eng Part A..

[69] Zhang X, Zhou Y, Li H, Wang R, Yang D, Li B, Fu J (2018). Intravenous administration of DPSCs and BDNF improves neurological performance in rats with focal cerebral ischemia. Int J Mol Med.

[70] Yamagata M, Yamamoto A, Kako E, Kaneko N, Matsubara K, Sakai K, Sawamoto K, Ueda M (2013). Human dental pulp-derived stem cells protect against hypoxic-ischemic brain injury in neonatal mice. Stroke.

[71] Hata M, Omi M, Kobayashi Y, Nakamura N, Tosaki T, Miyabe M, Kojima N, Kubo K, Ozawa S, Maeda H, Tanaka Y, Matsubara T, Naruse K (2015). Transplantation of cultured dental pulp stem cells into the skeletal muscles ameliorated diabetic polyneuropathy: therapeutic plausibility of freshly isolated and cryopreserved dental pulp stem cells. Stem Cell Res Ther.

[72] Almeida PN, Souza GT, de Souza CM, de Zanette RS, Maranduba CP, Rettore JV, de Santos MO, do Carmo AM, da Maranduba CM, de Silva FS (2015). Proposing the use of dental pulp stem cells as a suitable biological model of neurofibromatosis type 1. Childs Nerv Syst.

[73] Omi M, Hata M, Nakamura N, Miyabe M, Ozawa S, Nukada H, Tsukamoto M, Sango K, Himeno T, Kamiya H, Nakamura J, Takebe J, Matsubara T, Naruse K (2017). Transplantation of dental pulp stem cells improves long-term diabetic polyneuropathy together with improvement of nerve morphometrical evaluation. Stem Cell Res Ther.

[74] Askari N, Yaqhoobi MM, Shamsara M, Esmaeili-Mahani S (2015). Tetracycline regulated expression of OLIG2 gene in human dental pulp stem cells lead to mouse sciatic nerve regeneration upon transplantation. Neuroscience.

[75] Sugimura-Wakayama Y, Katagiri W, Osugi M, Kawai T, Ogata K, Sakaguchi K, Hibi H (2015). Peripheral nerve regeneration by secretomes of stem cells from human exfoliated deciduous teeth. Stem Cells Dev.

[76] Beigi MH, Ghasemi-Mobarakeh L, Prabhakaran MP, Karbalaie K, Azadeh H, Ramakrishna S, Baharvand H, Nasr-Esfahani MH (2014). In vivo integration of poly(ε-caprolactone)/gelatin nanofibrous nerve guide seeded with teeth derived stem cells for peripheral nerve regeneration. J Biomed Mater Res A.

[77] Petcu EB, Midha R, McColl E, Popa-Wagner CTV (2018). 3D printing strategies for peripheral nerve regeneration. Biofabrication.

[78] Venkat P, Shen Y, Chopp M, Chen J. Cell-based and pharmacological neurorestorative therapies for ischemic stroke. Neuropharmacology. 2018;15:134(Pt B):310–22. 10.1016/j.neuropharm.2017.08.036 PMC5832535 28867364

[79] Lindvall O, Kokaia Z (2006). Stem cells for the treatment of neurological disorders. Nature.

[80] Song M, Jue SS, Cho YA, Kim EC (2015). Comparison of the effects of human dental pulp stem cells and human bone marrow-derived mesenchymal stem cells on ischemic human astrocytes in vitro. J Neurosci Res.

[81] Cho YA, Kim DS, Song M, Bae WJ, Lee S, Kim EC (2016). Protein interacting with never in mitosis A-1 induces glutamatergic and GABAergic neuronal differentiation in human dental pulp stem cells. J Endod..

[82] Young FI, Telezhkin V, Youde SJ, Langley MS, Stack M, Kemp PJ, Waddington RJ, Sloan AJ, Song B (2016). Clonal heterogeneity in the neuronal and glial differentiation of dental pulp stem/progenitor cells. Stem Cells Int.

[83] Martens W, Bronckaers A, Politis C, Jacobs R, Lambrichts I (2013). Dental stem cells and their promising role in neural regeneration: an update. Clin Oral Investig.

[84] Kiraly M, Porcsalmy B, Pataki A, Kádár K, Jelitai M, Molnár B, Hermann P, Gera I, Grimm WD, Ganss B, Zsembery A, Varga G (2009). Simultaneous PKC and cAMP activation induces differentiation of human dental pulp stem cells into functionally active neurons. Neurochem Int.

[85] Davidson RM (1994). Neural form of voltage-dependent sodium current in human cultured dental pulp cells. Arch Oral Biol.

[86] Xiao L, Tsutsui T (2013). Human dental mesenchymal stem cells, and neural regeneration. Hum Cell.

[87] Xiao Li, Ide Ryoji, Saiki Chikako, Kumazawa Yasuo, Okamura Hisashi (2017). Human Dental Pulp Cells Differentiate toward Neuronal Cells and Promote Neuroregeneration in Adult Organotypic Hippocampal Slices In Vitro. International Journal of Molecular Sciences.

[88] Baraniak PR, McDevitt TC (2010). Stem cell paracrine actions, and tissue regeneration. Regen Med.

[89] Tucker KL (2002). Neurotrophins and the control of axonal outgrowth. Panminerva Med.

[90] Schinder AF, Poo M (2000). The neurotrophin hypothesis for synaptic plasticity. Trends Neurosci.

[91] Arthur A, Shi S, Zannettino AC, Fujii N, Gronthos S, Koblar SA (2009). Implanted adult human dental pulp stem cells induce endogenous axon guidance. Stem Cells.

[92] Blesch A, Grill RJ, Tuszynski MH (1998). Neurotrophin gene therapy in CNS models of trauma and degeneration. Prog Brain Res.

[93] Ishizaka R, Hayashi Y, Iohara K, Sugiyama M, Murakami M, Yamamoto T, Fukuta O, Nakashima M (2013). Stimulation of angiogenesis, neurogenesis, and regeneration by side population cells from dental pulp. Biomaterials.

[94] Matsushita Y, Ishigami M, Matsubara K, Kondo M, Wakayama H, Goto H, Ueda M, Yamamoto A (2017). Multifaceted therapeutic benefits of factors derived from stem cells from human exfoliated deciduous teeth for acute liver failure in rats. J Tissue Eng Regen Med.

[95] Nosrat IV, Smith CA, Mullally P, Olson L, Nosrat CA (2004). Dental pulp cells provide neurotrophic support for dopaminergic neurons and differentiate into neurons in vitro; implications for tissue engineering and repair in the nervous system. Eur J Neurosci.

[96] Mead Ben, Logan Ann, Berry Martin, Leadbeater Wendy, Scheven Ben A. (2014). Paracrine-Mediated Neuroprotection and Neuritogenesis of Axotomised Retinal Ganglion Cells by Human Dental Pulp Stem Cells: Comparison with Human Bone Marrow and Adipose-Derived Mesenchymal Stem Cells. PLoS ONE.

[97] Marlier Q, Verteneuil S, Vandenbosch R, Malgrange B (2015). Mechanisms and functional significance of stroke-induced neurogenesis. Front Neurosci.

[98] Nosrat IV, Widenfalk J, Olson L, Nosrat CA (2001). Dental pulp cells produce neurotrophic factors, interact with trigeminal neurons in vitro and rescue motoneurons after spinal cord injury. Dev Biol.

[99] Xu X, Warrington AE, Bieber AJ, Rodriguez M (2011). Enhancing CNS repair in neurological disease: challenges arising from neurodegeneration and rewiring of the network. CNS Drugs.

[100] Shen CY, Li L, Feng T, Li JR, Yu MX, Lu MX, Li H (2015). Dental pulp stem cells derived conditioned medium promotes angiogenesis in hindlimb ischemia. Tissue Eng Regener Med.

[101] Marchionni C, Bonsi L, Alviano F, Lanzoni G, Di Tullio A, Costa R, Montanari M, Tazzari PL, Ricci F, Pasquinelli G, Orrico C, Grossi A, Prati C, Bagnara GP (2009). Angiogenic potential of human dental pulp stromal (stem) cells. Int J Immunopathol Pharmacol.

[102] Bronckaers A, Hilkens P, Fanton Y, Struys T, Gervois P, Politis C, Martens W, Lambrichts I (2013). Angiogenic properties of human dental pulp stem cells. PLoS One.

[103] Yang JP, Liu HJ, Wang ZL, Cheng SM, Cheng X, Xu GL, Liu XF (2009). The dose-effectiveness of intranasal VEGF in treatment of experimental stroke. Neurosci Lett.

[104] Winderlich JN, Kremer KL, Koblar SA (2016). Adult human dental pulp stem cells promote blood-brain barrier permeability through vascular endothelial growth factor-a expression. J Cereb Blood Flow Metab.

[105] Iohara K, Zheng L, Wake H, Ito M, Nabekura J, Wakita H, Nakamura H, Into T, Matsushita K, Nakashima M (2008). A novel stem cell source for vasculogenesis in ischemia: subfraction of side population cells from dental pulp. Stem Cells.

[106] Snider WD, Lichtman JW (1996). Are neurotrophins synaptotrophins?. Mol Cell Neurosci.

[107] Lee HT, Chang HT, Lee S, Lin CH, Fan JR, Lin SZ, Hsu CY, Hsieh CH, Shyu WC (2016). Role of IGF1R(+) MSCs in modulating neuroplasticity via CXCR4 cross-interaction. Sci Rep.

[108] Chiu HY, Lin CH, Hsu CY, Yu J, Hsieh CH, Shyu WC (2017). IGF1R dental pulp stem cells enhanced neuroplasticity in hypoxia-ischemia model. Mol Neurobiol.

[109] Tham TN, Lazarini F, Franceschini IA, Lachapelle F, Amara A, Dubois-Dalcq M (2001). Developmental pattern of expression of the alpha chemokine stromal cell-derived factor 1 in the rat central nervous system. Eur J Neurosci.

[110] Cheng CM, Mervis RF, Niu SL, Salem N, Witters LA, Tseng V, Reinhardt R, Bondy CA (2003). Insulin-like growth factor 1 is essential for normal dendritic growth. J Neurosci Res.

[111] Tang F, Guo S, Liao H, Yu P, Wang L, Song X, Chen J, Yang Q (2017). Resveratrol enhances neurite outgrowth and synaptogenesis via sonic hedgehog signaling following oxygen-glucose deprivation/reoxygenation injury. Cell Physiol Biochem.

[112] Sánchez-Camacho Cristina, Bovolenta Paola (2009). Emerging mechanisms in morphogen-mediated axon guidance. BioEssays.

[113] Eleuterio E, Trubiani O, Sulpizio M, Di Giuseppe F, Pierdomenico L, Marchisio M, Giancola R, Giammaria G, Miscia S, Caputi S, Di Ilio C, Angelucci S (2013). Proteome of human stem cells from periodontal ligament and dental pulp. PLoS One.

[114] Li Z, Jiang CM, An S, Cheng Q, Huang YF, Wang YT, Gou YC, Xiao L, Yu WJ, Wang J (2014). Immunomodulatory properties of dental tissue-derived mesenchymal stem cells. Oral Dis.

[115] Sonoyama W, Liu Y, Yamaza T, Tuan RS, Wang S, Shi S, Huang GT (2008). Characterization of the apical papilla and its residing stem cells from human immature permanent teeth: a pilot study. J Endod.

[116] Ding G, Niu J, Liu Y (2015). Dental pulp stem cells suppress the proliferation of lymphocytes via transforming growth factor-β1. Hum Cell.

[117] Martinez VG, Ontoria-Oviedo I, Ricardo CP, Harding SE, Sacedon R, Varas A, Zapata A, Sepulveda P, Vicente A (2017). Overexpression of hypoxia-inducible factor 1 alpha improves immunomodulation by dental mesenchymal stem cells. Stem Cell Res Ther.

[118] Hong JW, Lim JH, Chung CJ, Kang TJ, Kim TY, Kim YS, Roh TS, Lew DH (2017). Immune tolerance of human dental pulp-derived mesenchymal stem cells mediated by CD4^+^CD25^+^FoxP3^+^ regulatory T-cells and induced by TGF-β1 and IL-10. Yonsei Med J.

[119] Sofroniew MV (2009). Molecular dissection of reactive astrogliosis and glial scar formation. Trends Neurosci.

[120] Zhang L, Zhao W, Li B, Alkon DL, Barker JL, Chang YH, Wu M, Rubinow DR (2000). TNF-alpha induced over-expression of GFAP is associated with MAPKs. Neuroreport.

[121] Balasingam V, Yong VW (1996). Attenuation of astroglial reactivity by interleukin-10. J Neurosci.

[122] Toft-Hansen H, Füchtbauer L, Owens T (2011). Inhibition of reactive astrocytosis in established experimental autoimmune encephalomyelitis favors infiltration by myeloid cells over T cells and enhances severity of disease. Glia.

[123] Shimizu K, Guo W, Wang H, Zou S, LaGraize SC, Iwata K, Wei F, Dubner R, Ren K (2009). Differential involvement of trigeminal transition zone and laminated subnucleus caudalis in orofacial deep and cutaneous hyperalgesia: the effects of interleukin-10 and glial inhibitors. Mol Pain.

[124] Edwards JP, Zhang X, Frauwirth KA, Mosser DM (2006). Biochemical and functional characterization of three activated macrophage populations. J Leukoc Biol.

[125] Nauta AJ, Raaschou-Jensen N, Roos A, Daha MR, Madsen HO, BorriasEssers MC, Ryder LP, Koch C, Garred P (2003). Mannose-binding lectin engagement with late apoptotic and necrotic cells. Eur J Immunol.

[126] Kigerl KA, Gensel JC, Ankeny DP, Alexander JK, Donnelly DJ, Popovich PG (2009). Identification of two distinct macrophage subsets with divergent effects causing either neurotoxicity or regeneration in the injured mouse spinal cord. J Neurosci.

[127] Miron VE, Boyd A, Zhao JW, Yuen TJ, Ruckh JM, Shadrach JL, van Wijngaarden P, Wagers AJ, Williams A, Franklin RJ, ffrench-Constant C (2013). M2 microglia and macrophages drive oligodendrocyte differentiation during CNS remyelination. Nat Neurosci.

[128] Casella GT, Bunge MB, Wood PM (2006). Endothelial cell loss is not a major cause of neuronal and glial cell death following contusion injury of the spinal cord. Exp Neurol.

[129] Lou J, Lenke LG, Ludwig FJ, O'Brien MF (1998). Apoptosis as a mechanism of neuronal cell death following acute experimental spinal cord injury. Spinal Cord.

[130] Nicola FD, Marques MR, Odorcyk F, Arcego DM, Petenuzzo L, Aristimunha D, VizueteA SEF, Pereira DP, Maurmann N, Dalmaz C, Pranke P, Netto CA (2017). Neuroprotector effect of stem cells from human exfoliated deciduous teeth transplanted after traumatic spinal cord injury involves inhibition of early neuronal apoptosis. Brain Res.

[131] Matsubara K, Matsushita Y, Sakai K, Kano F, Kondo M, Noda M, Hashimoto N, Imagama S, Ishiguro N, Suzumura A, Ueda M, Furukawa K, Yamamoto A (2015). Secreted ectodomain of sialic acid-binding Ig-like lectin-9 and monocyte chemoattractant protein-1 promote recovery after rat spinal cord injury by altering macrophage polarity. J Neurosci.

[132] Gerber HP, Dixit V, Ferrara N (1998). Vascular endothelial growth factor induces expression of the antiapoptotic proteins Bcl-2 and A1 in vascular endothelial cells. J Biol Chem.

[133] Ahmed Nel-M, Murakami M, Hirose Y, Nakashima M. Therapeutic potential of dental pulp stem cell secretome for Alzheimer's disease treatment: an in vitro study. Stem Cells Int 20162016:8102478. 10.1155/2016/8102478 PMC4923581 27403169

[134] Oltvai ZN, Milliman CL, Korsmeyer SJ (1993). Bcl-2 heterodimerizes in vivo with a conserved homolog, Bax, that accelerates programmed cell death. Cell.

[135] Green DR, Reed JC (1998). Mitochondria and apoptosis. Science.

[136] Aimetti M, Ferrarotti F, Gamba MN, Giraudi M, Romano F (2018). Regenerative treatment of periodontal intrabony defects using autologous dental pulp stem cells: a 1-year follow-up case series. Int J Periodontics Restorative Dent.

[137] Chen FM, Gao LN, Tian BM, Zhang XY, Zhang YJ, Dong GY, Lu H, Chu Q, Xu J, Yu Y, Wu RX, Yin Y, Shi S, Jin Y (2016). Treatment of periodontal intrabony defects using autologous periodontal ligament stem cells: a randomized clinical trial. Stem Cell Res Ther.

[138] Hidalgo San Jose Lorena, Stephens Phil, Song Bing, Barrow David (2018). Microfluidic Encapsulation Supports Stem Cell Viability, Proliferation, and Neuronal Differentiation. Tissue Engineering Part C: Methods.

[139] Gnanasegaran Nareshwaran, Govindasamy Vijayendran, Kathirvaloo Premasangery, Musa Sabri, Abu Kasim Noor Hayaty (2017). Effects of cell cycle phases on the induction of dental pulp stem cells toward dopaminergic-like cells. Journal of Tissue Engineering and Regenerative Medicine.

[140] Feitosa MLT, Sarmento CAP, Bocabello RZ, Beltrão-Braga PCB, Pignatari GC, Giglio RF, Miglino MA, Orlandin JR, Ambrósio CE (2017). Transplantation of human immature dental pulp stem cell in dogs with chronic spinal cord injury. Acta Cir Bras.

[141] Yang C, Li X, Sun L, Guo W, Tian W (2017). Potential of human dental stem cells in repairing the complete transaction of rat spinal cord. J Neural Eng.

